# Community pharmacists’ strategies and barriers in supporting medication adherence in Saudi Arabia: a cross-sectional study

**DOI:** 10.3389/fmed.2026.1962692

**Published:** 2026-10-20

**Authors:** Fahad Alzahrani, Faris S. Alnezary, Zikria Saleem, Masaad Saeed Almutairi

**Affiliations:** 1 Department of Pharmacy Practice, College of Pharmacy, Taibah University, Madinah, Saudi Arabia; 2 Department of Pharmacy Practice, College of Pharmacy, Qassim University, Qassim, Saudi Arabia

**Keywords:** community pharmacists, health literacy, medication adherence, patient-centered care, perceived barriers, Saudi Arabia, public health

## Abstract

**Background:**

Community pharmacists are well positioned to identify medication non-adherence and support appropriate medication use. However, evidence regarding their adherence-related activities and perceived barriers in Saudi Arabia remains limited.

**Objectives:**

This study examined community pharmacists’ self-reported strategies for identifying medication non-adherence and supporting medication use, their attitudes and perceived barriers, and factors associated with their engagement in these activities.

**Methods:**

A cross-sectional survey was conducted among licensed community pharmacists from all five regions of Saudi Arabia between September and November 2025. A structured questionnaire adapted from previously published instruments assessed identification and support strategies, attitudes, perceived barriers, and participant characteristics. Data were analyzed using descriptive statistics, principal component analysis, and multiple linear regression.

**Results:**

Of 681 pharmacists invited, 382 responded (56.1%), and 345 eligible and complete responses were analyzed. Common identification approaches included the combined MEMS-or-pill-count item (50.8% reported using it at least 75% of the time), assessment of patients’ medication response (50.1%), and review of refill records (47.2%). Frequently reported support strategies included promoting self-monitoring (59.7%), using patient-friendly labeling (58.8%), and simplifying medication regimens (57.7%). Major perceived barriers included lack of private consultation areas (49.6%), time constraints (45.8%), limited clinical information (44.1%), and poor patient health literacy (40.6%). In the adjusted analysis, working ≥40 h per week was associated with a lower support-strategy score, whereas employment in a chain pharmacy was associated with a higher score. No examined variable was significantly associated with the identification-strategy score.

**Conclusion:**

Community pharmacists reported using several practical adherence-related strategies while experiencing important structural and informational barriers. Workplace characteristics were associated with their reported support activities; however, the cross-sectional design does not permit causal inference or establish improvements in patient adherence or clinical outcomes.

## Introduction

Medication non-adherence remains a persistent challenge in healthcare systems worldwide, including countries in the Middle East (1). The World Health Organization estimated that approximately half of patients with chronic diseases do not take their medications as prescribed (2), and a recent review published two decades after that report indicates that progress remains limited (3). Medication non-adherence is associated with poorer disease control, preventable complications, increased healthcare utilization, and higher costs (4–9). These consequences emphasize the need for practical methods to identify non-adherence and support medication use in routine clinical practice.

Community pharmacists are well positioned to contribute because of their accessibility and frequent contact with patients (10–12). Common methods for identifying non-adherence include direct patient questioning, assessment of treatment response, review of prescription-refill records, pill counts, standardized self-report scales, and electronic monitoring. However, each method has limitations. Self-reported information may be affected by recall and social-desirability biases, refill records may be incomplete or inaccessible, standardized tools may be difficult to incorporate into routine workflow, and electronic monitoring can be costly or unavailable. Pharmacists may also support adherence through patient education, medication-regimen simplification, reminder systems, adherence aids, patient-friendly labeling, and follow-up. Although pharmacist-led interventions may improve adherence, their effects vary across settings, and the quality of the supporting evidence remains inconsistent (13, 14). Implementation may also be limited by workload, insufficient privacy, inadequate training, limited access to clinical information, and low patient health literacy (15, 16).

In Saudi Arabia, community pharmacy practice has traditionally focused on medication supply, although recent health system reforms aim to expand pharmacists’ patient-centered and clinical roles (17, 18). Nevertheless, the implementation of clinical services remains variable, and workforce, resource, and interprofessional barriers continue to affect service delivery (19). Khawagi et al. recently examined medication-adherence assessment and management among healthcare professionals in Saudi Arabia, most of whom were pharmacists. They reported frequent reliance on direct questioning and treatment-response assessment, alongside barriers involving workload, consultation time, and inadequate adherence-tracking systems (20). This study extends this evidence by focusing on community pharmacists and examining their self-reported identification and support strategies, attitudes, perceived barriers, and the demographic and workplace characteristics associated with their engagement. Therefore, this study aimed to examine community pharmacists’ self-reported strategies for identifying medication non-adherence and supporting medication use, assess their attitudes and perceived barriers, and identify factors associated with their engagement in these activities.

## Methods

### Study design and setting

A cross-sectional survey was conducted among licensed pharmacists working in community pharmacies across the five geographical regions of Saudi Arabia. The study was reported in accordance with the Strengthening the Reporting of Observational Studies in Epidemiology (STROBE) guidelines.

### Sampling technique and sample size

The minimum required sample size was calculated using the Raosoft online calculator. Based on an estimated population of 20,900 pharmacists in Saudi Arabia (21), a 95% confidence level, a 5% margin of error, and a 50% response distribution, the required sample was 378 participants. Participants were recruited using non-probability convenience sampling through four community pharmacy chains. Of the calculated target, 345 eligible and complete responses were included in the analysis, representing 91.3% of the planned sample. This shortfall may have modestly reduced statistical precision and power, particularly for categories with few participants.

### Study population

Eligible participants were licensed community pharmacists aged 18 years or older, holding at least a bachelor's degree in pharmacy, working in Saudi Arabia, and willing to provide informed consent. Pharmacy assistants, trainees, students, unlicensed personnel, and pharmacists not working in community pharmacy settings were excluded.

### Study instruments

A structured, self-administered questionnaire was developed by adapting items and domains from previously published studies examining pharmacists’ medication-adherence practices, attitudes, and perceived barriers (22–24). Specifically, identification items 14.1–14.3, all 19 adherence-support items (15.1–15.19), all seven attitude items (16.1–16.7), and all 13 perceived-barrier items (17.1–17.13) were adapted primarily from Mansoor et al. (22). wo additional identification items—use of a standardized adherence scale and use of MEMS or pill counts—were added for the present study, while the broader content concerning health-promotion practices and medication-adherence support was informed by Kotecki et al. (23) and Nichols-English and Poirier (24).

TWe modified terminology, demographic and workplace characteristics, training items, and descriptions of professional roles to reflect the Saudi community pharmacy context. We also changed the response format for the 19 adherence-support items from the dichotomous yes/no responses used in the source instrument to a five-point frequency scale ranging from never to always. The questionnaire included sections assessing strategies for identifying medication non-adherence, strategies for supporting medication use, attitudes toward adherence support, perceived barriers, previous training, interest in further training, and demographic and professional characteristics of the respondents. Supplementary File 1 provides the complete questionnaire. Supplementary Table S1 provides the Pattern Matrix and component-specific reliability results for the perceived barrier components.

Three independent experts in pharmacy practice and public health, two professors and one clinical pharmacist, reviewed the questionnaire for face and content validity, clarity, relevance, and suitability for the Saudi context. We then pretested the questionnaire with 13 community pharmacists to assess its comprehensibility, completion time, relevance, and technical functionality. We used feedback from experts and pretest participants to clarify the wording before distribution. The final analysis excluded the pretest responses.

### Study variables

The two primary outcomes were the identification and support strategy composite scores. The identification strategy score was calculated as the mean of five items assessing how often pharmacists used strategies to identify medication non-adherence. The support strategy score was calculated as the mean of 19 items assessing strategies used to support medication adherence.

We coded responses from 1 to 5, with higher values indicating more frequent use. Consequently, both composite scores ranged from 1 to 5, with higher scores indicating greater self-reported engagement in the corresponding activity. We retained the original five response categories when calculating composite scores. We combined categories only for descriptive presentation in the tables and figure.

We selected demographic and practice-related variables *a priori* based on previous literature and their practical relevance to community pharmacy services. These included gender, education level, country of academic qualification, professional experience, geographical region, weekly working hours, practice site location, pharmacy type, number of pharmacists per shift, weekly prescription volume, previous adherence-support training, and interest in further training. We examined attitudes and perceived barriers as separate descriptive and psychometric constructs and did not include them as independent variables in these models. We excluded age from the final regression models because it conceptually and statistically overlapped with professional experience.

### Data collection

Data were collected between September and November 2025 using an electronic questionnaire administered through Google Forms. Licensed community pharmacists were invited through direct email invitations coordinated with four Saudi pharmacy chains. Reminder messages were sent periodically to improve participation. The questionnaire required approximately 8–10 min to complete.

Responses were collected anonymously and stored in a secure electronic database. Submitted questionnaires were screened for eligibility and completeness before analysis. Of the 382 responses received, 37 were excluded because they were incomplete or did not meet the eligibility criteria, resulting in a final analytical sample of 345 participants.

### Statistical analysis

We analyzed the anonymized data using IBM SPSS Statistics version 27. We summarized demographic characteristics, adherence strategies, attitudes, and perceived barriers using descriptive statistics. Continuous-variable distributions were assessed using probability plots. Responses were recorded on a 5-point scale. For descriptive reporting, the two lowest and two highest categories were combined, while the midpoint was retained separately.‏

We calculated identification- and support-strategy composite scores by averaging responses to five and 19 items, respectively. Both scores ranged from 1 to 5, with higher scores indicating more frequent engagement.‏

We conducted a principal component analysis separately for attitude and barrier items. We assessed sampling adequacy using the Kaiser–Meyer–Olkin measure and Bartlett's test of sphericity. We retained components with eigenvalues greater than 1 and based retention on the inspection of the scree plot. Direct Oblimin rotation was used when more than one component was retained, and pattern loadings of ≥ 0.30 were considered salient. Items were assigned to the component with the highest loading, and conceptual interpretability was considered for cross-loading items. Internal consistency was assessed using Cronbach's alpha, with values ≥0.70 considered acceptable.

Multiple linear regression analyses examined factors associated with the two composite scores. Predictors were selected *a priori* based on previous literature and practical relevance. Attitudes and barriers were treated as separate constructs and were not prespecified as predictors. Age was excluded because of its overlap with professional experience. Unstandardized coefficients (B) with 95% confidence intervals were reported. Regression assumptions were assessed using residual plots, Durbin–Watson statistics, tolerance, and variance inflation factors. Model performance was evaluated using F statistics, R^2^, and adjusted R^2^. Statistical significance was defined as *p* < 0.05.

### Ethical considerations

This study was approved by the Scientific Ethics Committee of the College of Pharmacy at Taibah University, Madinah, Saudi Arabia, on 16 September 2025 (approval no. COPTU-REC-156-20250916).

The study followed the Declaration of Helsinki. Participation was voluntary, and all participants provided electronic informed consent before completing the questionnaire. No personally identifiable information was collected.

## Results

### Participant recruitment

We invited 681 community pharmacists to participate; 382 responded, for an initial response rate of 56.1%. After screening, 37 responses were excluded because they were incomplete or did not meet the eligibility criteria. The final analysis therefore included 345 participants, representing 50.6% of those invited and 91.3% of the calculated minimum sample of 378.

### Participant characteristics

Most participants were men (91.6%), and their mean age was 34.61 years (SD 7.03). Most held a bachelor's degree in pharmacy (81.7%), had obtained their academic qualification outside Saudi Arabia (66.7%), worked 40 h or more per week (83.5%), practiced in an urban location (68.4%), and worked in chain pharmacies (93.6%). Participants from the Western Region accounted for 47.0% of the sample. Overall, 63.2% reported previous adherence-support training, while 70.4% expressed interest in receiving further training (Table 1).

**Table 1:** Pharmacists’ sociodemographic and professional characteristics.

Characteristics	Frequency	%
Gender		
Male	316	91.6
Female	29	8.4
Age, years, mean (SD)	34.61 (7.03)	
Level of education		
Bachelor's degree (BSc)	282	81.7
Doctor of Pharmacy (PharmD)	58	16.8
Postgraduate (MSc, PhD)	5	1.4
Country of academic qualification		
Saudi Arabia	115	33.3
Overseas	230	66.7
Experience (years)		
< 1 year	21	6.1
1–5 years	68	19.7
6–10 years	86	24.9
11–15 years	91	26.4
> 15 years	79	22.9
Regions		
Central Region	80	23.2
Western Region	162	47.0
Eastern Region	41	11.9
Southern Region	51	14.8
Northern Region	11	3.2
Average number of hours worked per week		
< 40	57	16.5
≥ 40	288	83.5
Practice site location		
Urban	236	68.4
Rural	63	18.3
Suburban	46	13.3
Type of community pharmacy		
Independent pharmacy	22	6.4
Chain pharmacies	323	93.6
Number of pharmacists per shift		
One	102	29.6
Two	146	42.3
Three	59	17.1
Four or more	38	11.0
Number of prescriptions per week		
< 100 prescriptions	148	42.9
100–199 prescriptions	95	27.5
200–299 prescriptions	48	13.9
300–399 prescriptions	13	3.8
≥ 400 prescriptions	41	11.9
Trained to mentor patients on medication adherence		
No	85	24.6
Yes	218	63.2
Unsure	42	12.2
Seeking training in adherence support.		
No	49	14.2
Yes	243	70.4
Unsure	53	15.4

### Strategies for identifying medication non-adherence

Among the 345 pharmacists, the most frequently reported identification strategies were the combined MEMS-or-pill-count item, used at least 75% of the time by 50.8% of participants, and assessment of patients’ response to medication (50.1%). Exploring barriers to adherence (47.5%) and reviewing prescription-refill records (47.2%) were also frequently reported. Specific standardized tools, such as the Morisky scale, were used at least 75% of the time by 41.7% of respondents (Figure 1). Because MEMS and pill counts were assessed through a single item, their individual frequencies could not be determined.

**Figure 1:**
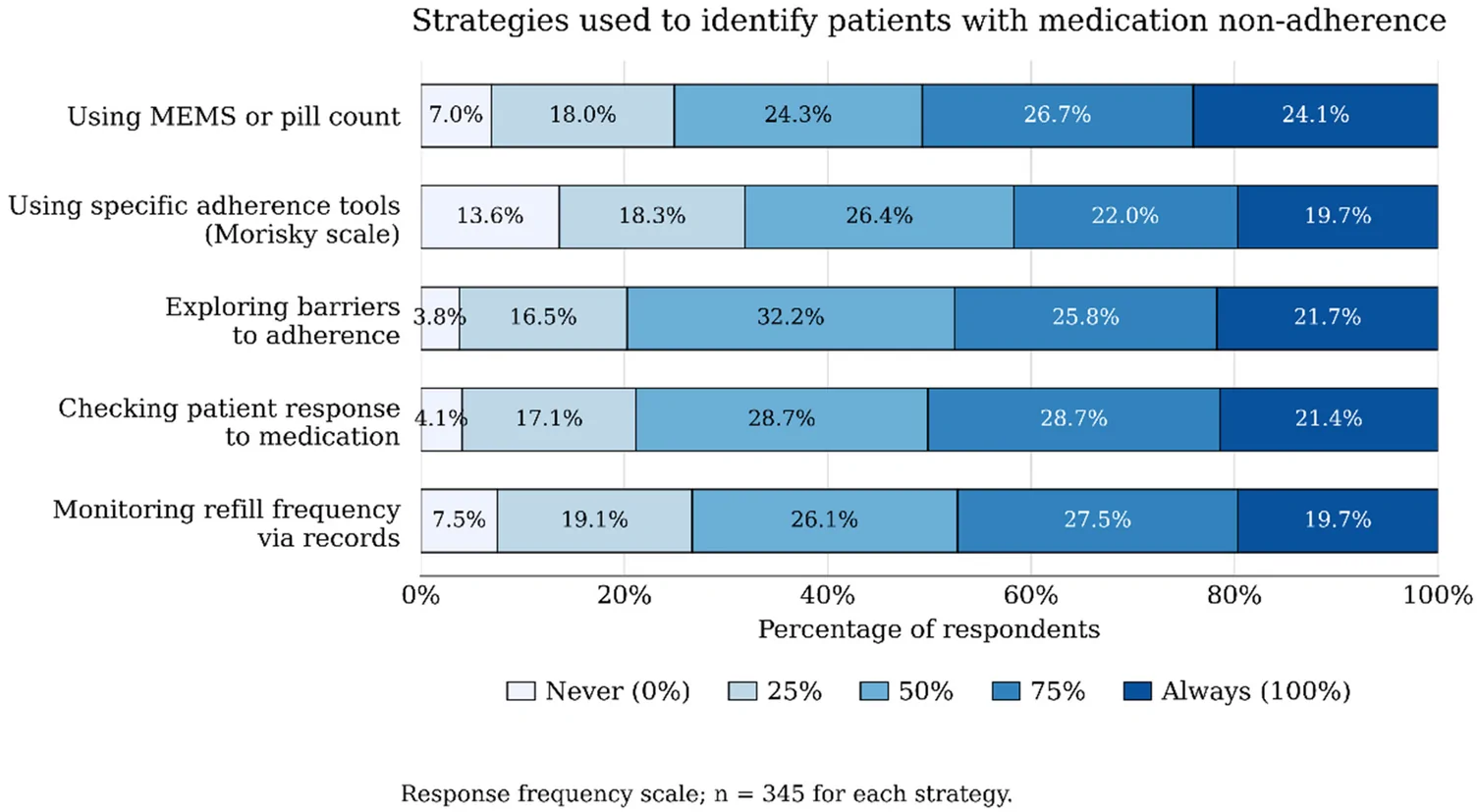
Distribution of self-reported strategies used by community pharmacists to identify patients with medication non-adherence (*n* = 345).

### Strategies for supporting medication adherence

The most frequently reported adherence-support strategies were promoting patient self-monitoring (59.7%), using labels in the patient's preferred language (58.8%), and simplifying medication regimens (57.7%). Providers reported linking medication use to daily activities (55.9%) and medication reviews (50.7%). Less frequently reported activities included providing dose-administration aids (45.8%), communicating with physicians about adherence (40.6%), and encouraging brand consistency (40.6%) (Table 2). Lung-function testing was reported as an indirect monitoring activity by 35.7% of respondents. This activity may help identify inadequate disease control and prompt discussion of medication use or inhaler technique, but it does not directly measure medication adherence.

**Table 2:** Strategies used by pharmacists to support patients’ adherence to medications.

Strategy	N/R	S	A/O
	n (%)	n (%)	n (%)
Providing dose administration aids (e.g., Webster-Pak)	51 (14.8)	136 (39.4)	158 (45.8)
Recommending medication review	55 (15.9)	115 (33.3)	175 (50.7)
Simplifying medication regimen	39 (11.3)	107 (31.0)	199 (57.7)
Linking medication to daily activities	48 (13.9)	104 (30.1)	193 (55.9)
Recommending a cheaper brand	48 (13.9)	113 (32.8)	184 (53.3)
Communicating with doctors about adherence	106 (30.7)	99 (28.7)	140 (40.6)
Encouraging brand consistency	93 (27.0)	112 (32.5)	140 (40.6)
Monitoring blood pressure	72 (20.9)	105 (30.4)	168 (48.7)
Encouraging self-monitoring	39 (11.0)	101 (29.3)	206 (59.7)
Recommending a medication change	126 (36.5)	93 (27.0)	126 (36.5)
Using labels in the patient's language	45 (13.0)	97 (28.1)	203 (58.8)
Monitoring blood glucose	70 (20.3)	102 (29.6)	173 (50.1)
Providing a medication calendar	89 (25.8)	103 (29.9)	153 (44.3)
Monitoring cholesterol	88 (25.5)	100 (29.0)	157 (45.5)
Using large-font labels	49 (14.2)	105 (30.4)	191 (55.4)
Sending SMS refill reminders	98 (28.4)	93 (27.0)	154 (44.6)
Following up on patient response (call/visit)	98 (28.4)	104 (30.1)	143 (41.4)
Conducting lung function tests	129 (37.4)	93 (27.0)	123 (35.7)
Sending letter refill reminders	105 (30.4)	102 (29.6)	138 (40.0)

N = Never, R = Rarely, S = Sometimes, O = Often, A = Always.

### Pharmacists’ attitudes toward adherence support

Principal component analysis of the 13 barrier items was appropriate, as indicated by a Kaiser–Meyer–Olkin value of 0.925 and a significant Bartlett's test of sphericity, *χ*^2^(78) = 2,639.344, *p* < 0.001. We retained two components with eigenvalues greater than 1. The first component had an eigenvalue of 7.054 and explained 54.26% of the variance, while the second had an eigenvalue of 1.073 and explained an additional 8.26%. Together, the two components explained 62.52% of the total variance. Direct Oblimin rotation converged after seven iterations, and the component correlation was 0.648. Based on the Pattern Matrix, the components were interpreted as role, stakeholder, and patient-related barriers (seven items; loadings 0.477–0.902; Cronbach's *α* = 0.908) and practice-resource and communication barriers (six items; loadings 0.458–0.843; Cronbach's *α* = 0.857). The complete 13-item barrier scale demonstrated excellent internal consistency (Cronbach's *α* = 0.939). Detailed rotated factor loadings, item assignments, and component-specific reliability estimates are presented in Supplementary Table S1.

Most pharmacists agreed that adherence education was part of their professional role (65.5%), that pharmacists should promote medication adherence (63.2%), and that all pharmacists should identify medication non-adherence (62.9%). More than half believed that physicians (55.1%) and patients (55.1%) supported pharmacists’ involvement. However, 49.6% agreed that adherence services were primarily the physician's responsibility (Table 3).

**Table 3:** Pharmacists’ attitudes and perceived barriers toward medication adherence support.

Items	SD/D, *n* (%)	Neutral, *n* (%)	SA/A, *n* (%)
Attitudes toward adherence support			
Pharmacists’ responsibility to identify non-adherence	33 (9.6)	113 (32.8)	199 (57.7)
Pharmacists’ role in promoting adherence	22 (6.4)	105 (30.4)	218 (63.2)
All pharmacists should identify non-adherence	24 (7.0)	104 (30.1)	217 (62.9)
Pharmacists’ role in adherence education	28 (8.1)	91 (26.4)	226 (65.5)
Doctors support pharmacists’ involvement	37 (10.7)	118 (34.2)	190 (55.1)
Patients view pharmacists as supporting adherence	32 (9.3)	123 (35.7)	190 (55.1)
Adherence service is the doctor's responsibility	46 (13.3)	128 (37.1)	171 (49.6)
Perceived barriers to adherence support			
Pharmacist-related barriers			
Lack of time	44 (12.8)	143 (41.4)	158 (45.8)
Lack of training in adherence services	66 (19.1)	137 (39.7)	142 (41.2)
Difficulty discussing adherence with patients	71 (20.6)	154 (44.6)	120 (34.8)
Difficulty discussing adherence with doctors	53 (15.4)	152 (44.1)	140 (40.6)
Perception that adherence is the doctor's role	86 (24.9)	154 (44.6)	105 (30.4)
Lack of clinical information	47 (12.2)	146 (42.3)	152 (44.1)
Lack of a private consultation area	42 (12.2)	132 (38.3)	171 (49.6)
Resistance from doctors	53 (15.4)	160 (46.4)	132 (38.3)
Lack of management support	114 (33.0)	126 (36.5)	105 (30.4)
Patient-related barriers			
Perception that adherence is not the pharmacist's role	99 (28.7)	130 (37.7)	116 (33.6)
Language barriers	95 (27.5)	127 (36.8)	123 (35.7)
Poor health literacy	64 (18.6)	141 (40.9)	140 (40.6)
Patient resistance to counseling	74 (21.4)	145 (42.0)	126 (36.5)

SD/D = strongly disagree/disagree; SA/A = strongly agree/agree.

### Perceived barriers to adherence support

Principal component analysis of the 13 barrier items was appropriate, as indicated by a KMO value of 0.925 and a significant Bartlett's test of sphericity, *χ*^2^(78) = 2639.344, *p* < 0.001. Two components with eigenvalues greater than 1 were retained. The first component had an eigenvalue of 7.054 and explained 54.26% of the variance, while the second had an eigenvalue of 1.073 and explained an additional 8.26%. Together, the two components explained 62.52% of the total variance. Item communalities ranged from 0.513 to 0.748, and Direct Oblimin rotation converged after seven iterations. The complete 13-item barrier scale demonstrated excellent internal consistency (Cronbach's *α* = 0.939).

The most frequently reported barriers were lack of a private consultation area (49.6%), lack of time (45.8%), limited clinical information (44.1%), insufficient training in adherence services (41.2%), and difficulty in discussing adherence with physicians (40.6%). Poor patient health literacy (40.6%), patient resistance to counseling (36.5%), and language barriers (35.7%) were also commonly reported (Table 3).

### Factors associated with identification and support strategies

The regression model for the identification-strategy score was statistically significant, F(27, 317) = 1.902, *p* = 0.005, explaining 13.9% of the variance in identification-strategy scores (R^2^ = 0.139; adjusted R^2^ = 0.066). However, none of the individual predictors was statistically significant at *p* < 0.05. Working with three pharmacists during a typical shift approached statistical significance (*B* = 0.35, 95% CI 0.00 to 0.70, *p* = 0.050). Postgraduate education (*B* = −0.92, 95% CI −1.85 to 0.01, *p* = 0.053) and PharmD education (*B* = −0.33, 95% CI −0.68 to 0.02, *p* = 0.067) were also not significantly associated with the identification-strategy score.

The regression model for the support-strategy score was statistically significant, F(27, 317) = 2.079, *p* = 0.002, explaining 15.0% of the variance in support-strategy scores (R^2^ = 0.150; adjusted R^2^ = 0.078). Working 40 h or more per week was associated with a lower support-strategy score (*B* = −0.30, 95% CI −0.56 to −0.04, *p* = 0.026), whereas working in a chain pharmacy was associated with a higher score (B = 0.56, 95% CI 0.16 to 0.96, *p* = 0.006). We found no other statistically significant associations (Table 4).

**Table 4:** Multiple linear regression of the association between identification and support strategy scores and potential predictor variables.

Variables	Identification strategy score			Support Strategy		
	B	95% CI*	*p-*value	B	95% CI*	*p*-value
**Gender**						
Male	Reference			Reference		
Female	0.174	−0.301 to 0.649	0.472	−0.172	−0.587 to 0.244	0.417
**Level of education**						
Bachelor's degree (BSc)	Reference			Reference		
Doctor of Pharmacy (PharmD)	−0.331	−0.684 to 0.023	0.067	0.074	−0.235 to 0.383	0.638
Postgraduate (MSc, PhD)	−0.921	−1.852 to 0.010	0.053	−0.545	−1.359 to 0.269	0.189
**Country of academic qualification**						
Saudi Arabia	Reference			Reference		
Overseas	−0.061	−0.362 to 0.239	0.689	0.083	−0.179 to 0.346	0.533
**Experience (years)**						
< 1 year	Reference			Reference		
1–5 years	0.336	−0.224 to 0.896	0.238	0.260	−0.230 to 0.749	0.298
6–10 years	0.411	−0.186 to 1.008	0.176	0.498	−0.024 to 1.020	0.061
11–15 years	0.301	−0.310 to 0.911	0.333	0.254	−0.279 to 0.788	0.349
> 15 years	0.188	−0.424 to 0.800	0.545	0.130	−0.405 to 0.665	0.634
**Regions**						
Central Region	Reference			Reference		
Western Region	−0.099	−0.380 to 0.181	0.487	−0.154	−0.399 to 0.092	0.218
Eastern Region	−0.087	−0.478 to 0.303	0.661	−0.201	−0.543 to 0.141	0.248
Southern Region	0.105	−0.263 to 0.473	0.576	0.081	−0.241 to 0.402	0.623
Northern Region	−0.632	−1.300 to 0.036	0.064	−0.114	−0.699 to 0.470	0.700
**Average number of hours worked per week**						
< 40	Reference			Reference		
≥ 40	−0.166	−0.464 to 0.133	0.276	−0.297	−0.558 to −0.036	0.026
**Practice site location**						
Urban	Reference			Reference		
Rural	−0.004	−0.300 to 0.293	0.980	0.059	−0.200 to 0.319	0.653
Suburban	0.207	−0.118 to 0.532	0.211	0.057	−0.227 to 0.341	0.693
**Type of community pharmacy**						
Independent pharmacy	Reference			Reference		
Chain pharmacies	0.349	−0.108 to 0.807	0.134	0.558	0.158 to 0.958	0.006
**Number of pharmacists working at the pharmacy during a typical shift**						
One	Reference			Reference		
Two	0.089	−0.182 to 0.359	0.519	0.067	−0.170 to 0.304	0.579
Three	0.352	0.000 to 0.704	0.050	0.284	−0.024 to 0.592	0.071
Four or more	−0.026	−0.419 to 0.368	0.898	−0.078	−0.422 to 0.266	0.656
**No. of prescriptions per week**						
< 100 prescriptions	Reference			Reference		
100–199 prescriptions	−0.192	−0.465 to 0.080	0.167	−0.007	−0.246 to 0.231	0.952
200–299 prescriptions	−0.089	−0.434 to 0.255	0.611	−0.101	−0.402 to 0.201	0.512
300–399 prescriptions	0.263	−0.331 to 0.857	0.384	0.223	−0.296 to 0.743	0.398
≥ 400 prescriptions	0.046	−0.331 to 0.423	0.810	0.253	−0.076 to 0.583	0.132
**Trained to mentor patients on medication adherence**						
No	Reference			Reference		
Yes	0.231	−0.044 to 0.505	0.099	0.227	−0.013 to 0.467	0.064
Unsure	0.017	−0.386 to 0.419	0.935	0.033	−0.319 to 0.384	0.855
**Seeking training in mentoring and supporting patients’ medication adherence.**						
No	Reference			Reference		
Yes	−0.002	−0.324 to 0.321	0.992	0.170	−0.112 to 0.452	0.237
Unsure	−0.379	−0.807 to 0.049	0.082	−0.052	−0.426 to 0.322	0.784

B = unstandardized regression coefficient; CI = confidence interval; Ref. = reference category. Bold values indicate *p* < 0.05.

The Durbin–Watson statistics were 1.890 for the identification model and 1.877 for the support model. VIF values ranged from 1.114 to 6.600, indicating no severe multicollinearity based on the prespecified VIF threshold of 10.

## Discussion

This study examined community pharmacists’ self-reported strategies for identifying and supporting medication adherence, together with their attitudes, perceived barriers, and associated demographic and workplace characteristics. Pharmacists reported using several practical identification and support strategies and generally expressed positive attitudes toward their professional role. Nevertheless, they identified important organizational, informational, and patient-related barriers. In the adjusted analyses, employment in a chain pharmacy was associated with higher support-strategy scores, whereas working 40 h or more per week was associated with lower scores. No examined characteristic was significantly associated with the identification-strategy score.

Pharmacists commonly reported assessing patients’ responses to medications, exploring adherence barriers, and reviewing prescription-refill records. These approaches are practical in community pharmacy settings and may facilitate direct discussions with patients about medication-taking difficulties (25, 26). However, the survey did not determine how pharmacists linked refill records to individual patients or used such records to assess adherence.

Standardized adherence-assessment tools, such as the Morisky scale, were reported less frequently, possibly because of time constraints, training needs, access considerations, and difficulty incorporating these instruments into routine workflow. However, the survey did not assess pharmacists’ access to licensed or validated versions of these tools; therefore, no conclusions can be drawn regarding the reasons for their limited reported use.

The combined questionnaire item referring to “MEMS or pill counts” was frequently endorsed. Because these methods were assessed in a single item, the findings cannot distinguish the use of electronic monitoring from pill counts or determine how either method was performed and interpreted. Consequently, the reported percentage should not be considered evidence of widespread use of electronic adherence-monitoring systems. Future studies should assess these methods separately and examine how they are implemented in routine practice.

Frequently reported support strategies included encouraging self-monitoring, simplifying medication regimens, using large-font or native-language labels, and linking medication-taking to daily activities. These patient-centered approaches are feasible within community pharmacy practice and may help patients incorporate medication use into their everyday routines (12, 28, 29). Lung-function testing was reported less frequently and should not be interpreted as a direct measure of adherence. When clinically appropriate, it may provide information about disease control or treatment response and prompt further assessment of adherence, inhaler technique, or the need for referral.

Pharmacists generally considered adherence support part of their professional role. However, limited consultation privacy, insufficient time, inadequate access to clinical information, poor patient health literacy, and patient resistance to counseling were commonly reported barriers. These findings are consistent with those of Khawagi et al., who identified workload, consultation-time limitations, and inadequate adherence-tracking systems among healthcare professionals in Saudi Arabia, most of whom were pharmacists (20). They also align with broader Saudi evidence concerning barriers to implementation of expanded community-pharmacy services (27). The present study adds community-pharmacy-specific evidence by examining reported identification and support strategies alongside workplace characteristics and perceived barriers.

The regression findings represent associations and should not be interpreted as causal effects. Employment in a chain pharmacy was associated with higher support-strategy scores, which may reflect differences in organizational procedures, staffing structures, or access to standardized resources (19); however, these mechanisms were not directly examined. Conversely, working 40 h or more per week was associated with lower support-strategy scores, possibly because workload and time pressures limit opportunities for individualized counseling (15, 16). Longitudinal and organizational-level studies are needed to investigate these possible explanations.

Education level, previous adherence-support training, and interest in further training were not significantly associated with either outcome. These findings do not demonstrate that training increases pharmacists’ engagement in adherence-related activities. Nevertheless, insufficient training was commonly perceived as a barrier, indicating a possible discrepancy between pharmacists’ perceived needs and the associations observed in the regression analysis.

Overall, improving adherence support may require attention to workplace conditions as well as professional development. Protected counseling time, appropriate consultation areas, access to clinical information, clear referral pathways, and practical adherence-assessment resources may support more consistent service delivery. Future research should distinguish between pharmacists’ familiarity with adherence tools and their routine use and should evaluate whether reported strategies are associated with improvements in patient adherence and clinical outcomes.

### Limitations

This study has several limitations that should be acknowledged. First, the cross-sectional design precludes establishing causal relationships between pharmacist characteristics and their engagement in adherence support. Second, the reliance on convenience sampling may limit the generalizability of the findings to the broader population of community pharmacists in Saudi Arabia, although the sample spanned all five regions. Gender imbalance in the pharmacist workforce and in study samples has been identified as a persistent, field-wide issue in Saudi community pharmacy research rather than one unique to this study (19). Third, the use of a self-administered questionnaire introduces the potential for social desirability and recall biases, as participants may overreport positive practices. Finally, the high reported use of MEMS (50.8%) may be an overestimation; respondents may have conflated this advanced technology with standard pill counts or computerized refill tracking, as true MEMS are rarely utilized in routine community pharmacy settings.

## Conclusion

This study highlights the important role of community pharmacists in promoting medication adherence within the Saudi healthcare system, aligning with the broader clinical mandates of the Saudi Vision 2030 Health Sector Transformation Program. Pharmacists reported using a range of practical strategies to identify and support non-adherent patients, but they also faced persistent barriers, including time constraints, insufficient training, limited infrastructure, and patient-related challenges. The findings suggest that organizational context and workload may influence pharmacists’ engagement in adherence-support activities, but causal relationships cannot be inferred from this cross-sectional study. Addressing these challenges through policy reform, professional development, protected time, private counseling spaces, and enhanced interdisciplinary collaboration may strengthen pharmacists’ contribution to medication adherence and patient outcomes.

## Data Availability

The raw data supporting the conclusions of this article will be made available by the authors, without undue reservation.
